# Supplementary material for: An open-source computational and data resource to analyze digital maps of immunopeptidomes
Source: eLife. 2015 Jul 8;4:e07661. doi: 10.7554/eLife.07661 (PMC4507788; doi:10.7554/eLife.07661)
Supplement: Supplementary file 1. — Description of the Python and the R scripts for the automated annotation and visualization of HLA peptidomic data. DOI: http://dx.doi.org/10.7554/eLife.07661.029 [file elife07661s008.pdf]

Supplementary file 1

Automated annotation and visualization of HLA peptidomic data

The set of software tools described below enables automated annotation and visualization of HLA peptidomic data. Python and R were used to write the scripts.

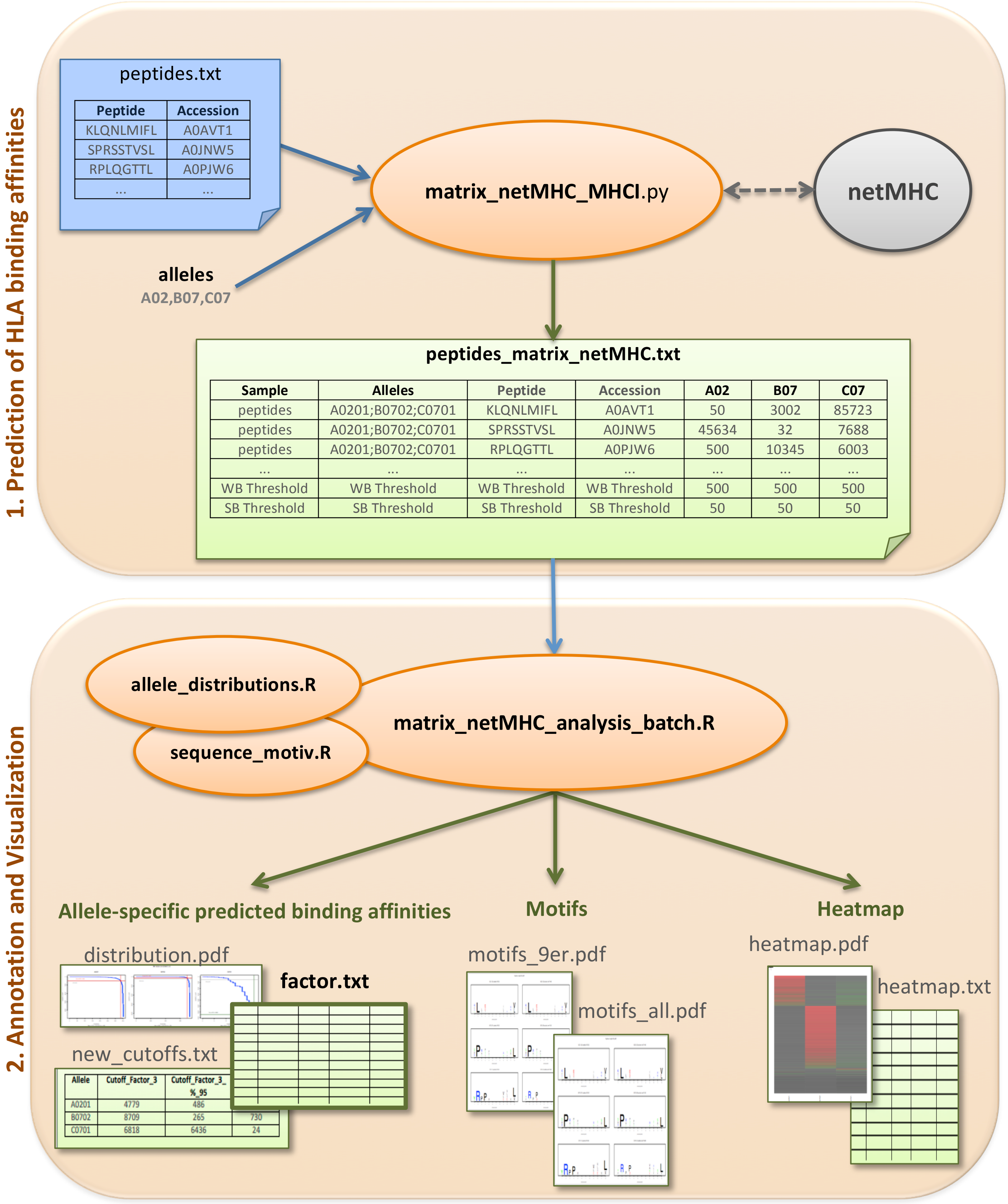

1. Prediction of HLA binding affinities

From a list of peptides identified from an HLA-typed biological sample, we obtain a new table including the **binding affinity predicted by NetMHC 3.4 for each HLA allele expressed in the sample**. This netMHC matrix output is then used in the next step to automatically annotate the peptides to their respective HLA allele. The output file also includes the original affinity thresholds used for weak binders (500 nM) and strong binders (50 nM) (two last rows).

The length of the peptides should be between **8 and 12** amino acids, otherwise they will be filtered out. **Duplicated** peptide sequences will be reported only once and the protein accession IDs will be combined using a semicolon.

Peptide **modifications** (e.g., oxidation) are usually ignored and their naked sequences are used in the output.

In case no allele selection is provided, a prediction will be done for all NetMHC-available alleles.

```
$ head peptides_test.txt
   Peptide    Accession
   KLQNLNIFL  A0AVT1
   SPRSSTVSL  A0JNW5
   RPLQGTTL   A0PJW6
   ...

$ matrix_netMHC_MHCI.py peptides_test.txt A02,B07,C07

   0. Parameters, input files and format handling:
        - Input file: peptides_test.txt
        - Output file: peptides_test_matrix_netMHC.txt

        - Fixing input file to ensure linux end of lines
              - Executing: perl -pi -e 's/\r\n/\n/g' peptides_test.txt
              - Executing: perl -pi -e 's/\r/\n/g' peptides_test.txt

    1. Match the given alleles to the netMHC available ones ...
        - Alleles to predict selected (3): A0201, B0702, C0701
     ...DONE!

   2. Parsing the input table ...
        - Peptide length allowed: [8 - 12]
              - Read 26 input lines, including 0 duplicates
        - Found 26 unique peptides: ILDFQPPEL, SPRAPFYRPL, LILMGNALIL... TMADQIVTV
     ...DONE!

   3. Processing peptides with netMHC...
     ...DONE!

   4. Write output file...
        - Written 26 peptide rows to peptides_test_matrix_netMHC.txt
        - Done, added 2 threshold rows to peptides_test_matrix_netMHC.txt
     ...DONE!

$ cat peptides_test_matrix_netMHC.txt
   Sample        Alleles            Peptide       Accession     A0201   B0702  C0701
   peptides_test  A0201;B0702;C0701 ILDFQPPEL     A4D174        32      19503  6355
   peptides_test  A0201;B0702;C0701 SPRAPFYRPL    A6NED2        27294   9      22048
   ...
   WB Threshold   WB Threshold      WB Threshold  WB Threshold  500     500    500
   SB Threshold   SB Threshold      SB Threshold  SB Threshold  50      50     50
```

2. Annotation and Visualization

The R script ‘**matrix_netMHC_analysis_batch.R**’ annotates the peptides, generates the heatmap and plot the distributions of the predicted binding affinities for the annotated HLA allele-specific peptides.

It needs to call functions from two other R scripts: ‘**allele_distributions.R**’ and ‘**sequence_motiv.R**’.

The script performs the following analysis steps:

- First a value called **‘factor/annotation score’** is calculated as the fold change between the best and the second best affinity value. The higher the annotation score is, the more confident the annotation of a peptide to an allele will be. The minimum to be considered in the current study as a valid annotation is 3, independently of the affinity value. The column ‘factor/annotation score’ is added to the original table and written to the output table. This value can be changed in future studies in the R code, variable ‘FACTOR_TH_MIN’. By computing an annotation score, even peptides with a predicted affinity not passing the NetMHC weak binder threshold (500 nM) can be confidently annotated to an allele when the predicted affinity values for the other HLA alleles are significantly worse. Non-annotated peptides with a score below 3 are curated in the output file and correspond to 1) non-HLA peptides/contaminants, 2) peptides predicted to strongly bind more than one HLA allele (supertype peptides), 3) exceptional HLA peptides with no known binding motifs. **The next version of the software tools will integrate statistical bootstrapping analysis to determine the optimal annotation cutoff value for individual datasets. The next upgrade will also integrate additional epitope prediction algorithms from IEDB and will annotate supertype peptides. We also plan to integrate a predictor-independent strategy [e.g. alignment- and clustering-based approach similar to GibbsCluster and NNalign (Andreatta et al. 2013; Nielson et al. 2009)] since the HLA annotation score used in this manuscript can only be calculated for well-characterized HLA alleles.**
- Optionally (set variable ‘DO_MOTIFS’) it displays the **motifs** of the peptides assigned to each allele using the new and the standard weak binder netMHC cutoff. Two PDF files are generated, one containing all lengths and other just for the 9-mers.
- The last step is to visualize the HLA annotation results on a **heatmap** (PDF file). The heatmap values are also stored in a tab-separated file.

The alleles with **no peptide annotated** will be removed from the outputs (except heatmap which will show everything).

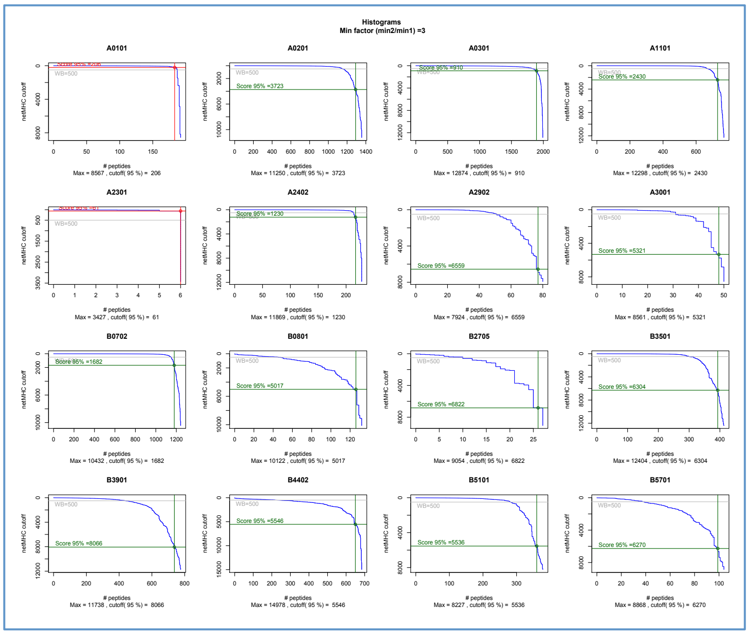

Distribution of predicted binding affinities from a list of annotated HLA allele-specific peptides

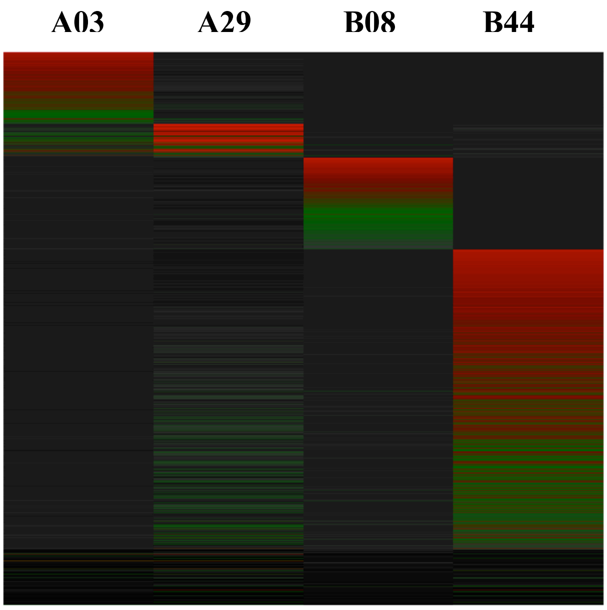

Heat map representation of an HLA peptidome from a B-lymphoblastoid cell line

The software tool uses Rscript to run in batch mode (not interactively), like a normal command line program in any machine with R installed. Command is described below.

```
$ Rscript $R_MHCI_SCRIPTS/matrix_netMHC_analysis_batch.R
"input_file=\"peptides_test_matrix_netMHC.txt\""
"output_file=\"peptides_test_matrix_netMHC_factor.tsv\""

     [1] " - Parameters: * input_file: ' peptides_test_matrix_netMHC.tsv '"
     [1] "               * output_file: ' peptides_test_matrix_netMHC_factor.tsv '"
     [1] " - Weak Binder Threshold: 500"
     [1] " - Calculating minimum and factors..."
     [1] "    * Writing factor to output file: 'peptides_test_matrix_netMHC_factor.tsv'"
     [1] " - Calculating new affinity cutoffs (95%)..."
     [1] "    * Minimum factor: 3"
     [1] "    * Printing distributions to PDF file:'peptides_test_allele_distributions.pdf'"

           Allele Cutoff_Factor_3 Cutoff_Factor_3_%_95 num_peps
     A0201  A0201            4779                  486      353
     B0702  B0702            8709                  265      730
     C0701  C0701            6818                 6436       24

     [1] "    * Output: factor_df_file: 'peptides_test_new_netMHC_cutoff_3.tsv'"
     [1] " - Displaying motifs..."
     [1] "    * Printing all length motifs to PDF file: 'peptides_test_motifs_all.pdf'"
     [1] "    * Printing length 9 motifs to PDF file: 'peptides_test _motifs_9ers.pdf'"
     [1] " - Creating heatmap..."
     [1] "    * Writing heatmap table to file: 'peptides_test_heatmap_table.tsv'"
     [1] "    * Printing heatmap to PDF file: 'peptides_test_heatmap_Rplot.pdf'"
     [1] "    * Classification of the peptides: A0201(353), B0702(730), C0701(24),
                                                UNCLASSIFIED(268)”
```

The following source data is available for Supplementary file 1:

Source Code 1. Python and R scripts: allele_distributions.R; matrix_netMHC_analysis_batch.R; matrix_netMHC_MHCI.py; sequence_motiv.R

REFERENCES (Supplementary file 1):

Andreatta, M., Lund, O., and Nielsen, M. (2013) Simultaneous alignment and clustering of peptide data using a Gibbs sampling approach. *Bioinformatics* **29**: 8–14

Nielsen, M., and Lund, O. (2009) NN-align. An artificial neural network-based alignment algorithm for MHC class II peptide binding prediction. *BMC Bioinformatics* **10**: 296
